# Hospital-Acquired Pneumonia in Patients Undergoing Coronary Artery Bypass Graft; Comparison of the Center for Disease Control Clinical Criteria With Physicians’ Judgment

**DOI:** 10.5812/aapm.20733

**Published:** 2014-08-17

**Authors:** Mahboubeh Baghban, Omalbanin Paknejad, Fardin Yousefshahi, Keivan Gohari Moghadam, Payvand Bina, Saghar Samimi Sadeh

**Affiliations:** 1 Internal Medicine Department, Faculty of Medicine, Tehran University of Medical Sciences, Tehran, Iran; 2 Anesthesia and Critical Care Department, Faculty of Medicine, Tehran University of Medical Sciences, Tehran, Iran; 3 Department of Basic and Clinical Research, Tehran Heart Center, Tehran University of Medical Sciences, Tehran, Iran

**Keywords:** Hospital Infection, Pneumonia, Coronary Artery Bypass, Graft

## Abstract

**Background:**

Following coronary artery bypass graft (CABG), patients are at high risk (3.2%-8.3%) for developing hospital-acquired pneumonia (HAP) with mortality rate of 24% to 50%. Some of routine features in patients undergoing CABG are similar to clinical criteria of Center of Disease Control (CDC) for diagnosis of pneumonia. This may lead to over-diagnosis of pneumonia in these patients.

**Objectives:**

This study aimed to assess the frequency of CDC criteria for diagnosis of pneumonia in patients undergoing CABG.

**Patients and Methods:**

This study was performed on CABG candidates admitted to post cardiac surgery Intensive Care Unit (ICU) in a six-month period. Patient’s records, Chest-X-Ray, and Laboratory tests were assessed for PNU1-CDC criteria for HAP diagnosis. At the same time, a physician who was unaware of the study protocol assessed the clinical diagnosis. Then the results were compared with CDC criteria-based diagnosis.

**Results:**

Of total 300 patients, 9 (3%) met CDC criteria for diagnosis of pneumonia while none of the cases were diagnosed as HAP according to the physicians’ clinical diagnosis. All nine patients were discharged with proper general condition and no need of antibiotic therapy. This study showed that loss of consciousness, tachypnea, dyspnea, PaO2 < 60 mm Hg, PaO2/FiO2 < 240, and local infiltration in 24 hours of operation were misleading features of CDC criteria, which were not considered in physicians’ clinical judgment to establish the diagnosis.

**Conclusions:**

Our findings suggest that in Post-CABG patients, physicians could judge the occurrence of HAP more accurately in comparison to making the diagnosis based on CDC criteria alone. Expert physician may intentionally do not take some of these criteria into account according the patients’ course of disease. Therefore, it is suggested that the value of these criteria in special group of patients like those undergoing CABG should be re-evaluated.

## 1. Background

Hospital-acquired pneumonia (HAP) is characterized by presentation of pneumonia at least 48 hours following hospital admission. Ventilator-associated pneumonia (VAP), which comprises about 80% of HAPs, occurs in 48 hours of intubation (1, 2).

Prompt diagnosis and treatment of HAP in these patients is accompanied by reduced mortality and hospitalization (3-6). Currently, there are two approaches for diagnosis of HAP: quantitative culture of the pathogen and clinical approach based on findings of studies on quantitative culture. Recent Infectious Disease Society of America (IDSA)/American Thoracic Society (ATS) guidelines suggest that both approaches are appropriate (7). Center for Disease Control (CDC) criteria for the diagnosis of pneumonia, which only take clinical characters into account (PNU1), includes (8):

1. Signs and symptoms:
  - Body temperature > 38℃ without known cause
  - Leukocytosis > 12,000, leucopenia < 4000/μL, or band cell > 50%
  - Mental confusion without known cause in patients aged more than 70 years
2. Other signs and symptoms:
  - New-onset purulent sputum, change in the quality of the sputum, increase in the respiratory secretions, or augmentation of the frequency of suctioning
  - New-onset or worsening of the cough, dyspnea, or tachypnea
  - Rale or bronchial respiratory sounds
3. Chest-X-ray (CXR):
  - New or persistent progressive infiltration
  - Consolidation
  - Cavitation

For diagnosis of pneumonia, patients should attain at least one score from part one, at least two scores form part two, and at least one score form part three (1, 7, 8).

Clinical presentation of VAP is similar to other forms of pneumonia and consists of fever, leukocytosis, increased respiratory secretions, and lung consolidation in physical examination along with the evidence of new-onset or progressive radiographic infiltration. In comparison with non-intubated subjects, higher frequency of abnormal findings in CXR of intubated patients and technical limitations of portable radiography makes the interpretation of the radiography more complicated in this group of patients. Other clinical characteristics are tachypnea, tachycardia, worsening of the oxygenation, and increased minute ventilation (9). 

Patients who undergo coronary artery bypass graft surgery (CABG) are at higher risk for VAP (3.2%-8.3%) and have worse prognosis (9-13). VAS pertains to patients undergoing CABG who need more than 48 hours of ventilation with mortality rate of 24%-50% (9, 14-17). VAP diagnosis in CABG patients could be difficult due to high frequency of fever and leukocytosis as well as difficulties in interpretation of abnormal findings in CXR after CABG (18-20). Therefore, it seems that there are no precise criteria for distinguishing pneumonia from other noninfectious etiologies of the lung infiltrations in Post-CABG phase.

Similarity of frequent signs and symptoms in post-CABG patients and CDC criteria for diagnosis of HAP may lead to overdiagnosis of HAP in this group of patients. Therefore, it may cause overprescription of antibiotics, which in turn can endanger patients by adverse effect of antibiotics, and enhance the risk of emergence of antibiotic-resistance pathogens, unnecessary diagnostic procedures, and treatment costs.

## 2. Objectives

The aim of this study was to assess the frequency of CDC clinical criteria for diagnosis of pneumonia in a selected group of patients undergoing CABG and to compare them with physicians’ clinical judgment in order to assess the validity of criteria in special circumstances.

## 3. Patients and Methods

This study was a case series approved by the Deputy of Research of Tehran University of Medical Sciences and included 300 patients undergoing CABG for about a six-month period starting in September 2011. All patients who met the inclusion criteria were included. All the patients who underwent CABG with extracorporeal cardiopulmonary pump in Tehran Heart Center were enrolled. Inclusion criteria were carbon dioxide pressure (CO_2_) < 45 mmHg and pressure of arterial oxygen (PaO_2_) > 60 mmHg, forced expiratory volume in first second (FEV1) > 60%, ejection fraction (EF) > 30%, and body mass index (BMI) < 40 kg/m^-2^. Patients with the following conditions were excluded: CABG with other concurrent surgeries, intubation in operation room, preoperative inotrope therapy, hemodynamic impairment and/or aortic balloon pump preoperatively or during first three hours of entrance to the ICU, corticosteroids or antibiotics use prior to the surgery, other heart pathology such as severe valve or congenital diseases, no infection prior to the surgery according to patient's history, clinical examination, and paraclinical assessments including CXR, severe renal insufficiency necessitating dialysis, and respiratory distress or evident respiratory problems before surgery. In first hours after entrance to the ICU, all patients were evaluated according to the eligibility criteria (11, 21).

Patients with unexpected surgical intervention of heart valves during surgery, re-operation after extubation, prolonged intubation in ICU (> 48 hours), those who received inotropic medication or had any documented loss in ejection fraction (defined as need for postoperative inotrope administration, intra-aortic balloon pump, a decrease in EF to ≥ 30, significant abnormalities in cardiac diastolic function, and/or clinical or paraclinical signs or symptoms of heart failure), those with any adverse neurological disorder (i.e. cerebrovascular accidents) or death during the first 48 hours postoperatively were also excluded.

Other variables included age, height, weight, EF before surgery, arterial blood gas (ABG), duration of intubation, CXR findings, and mortality during hospitalization. All patients were evaluated for postoperative respiratory complications by trained staff in the ICU for collecting the most relevant information.

All patients were admitted at least 72 hours prior to the surgery for preoperative assessments and other preparations. General anesthesia was attained according to a common protocol with relatively similar techniques and medications. Following anesthesia, expansion of atelectatic lungs was done in all patients prior to the sternum closure by performing “recruitment maneuver”. All patients were transferred to ICU when ventilation was maintained manually by ambo-bags. Patients’ head were elevated 30 degrees if no contraindication existed. Following awakens and stabilizing hemodynamics of the patient, extubation was performed and patients were evaluated for signs of HAP in the 24 to 48 hours following surgery. Respecting various aspects of "Open Lung Concept", ventilation and extubation was performed by expert physician and nurses (22).

After intubation, patients were ventilated by Drager anesthesia machine ventilator in operation room and by Drager ErITC2 ventilator in ICU. A checklist of demographic characteristics and clinical sings including age, sex, weight, height, body temperature, increase in respiratory secretions, new-onset purulent sputum, change in the quality (texture, color, or odor) of the sputum, augmentation of the frequency of suctioning, new-onset cough, worsening of the cough, dyspnea, loss of consciousness in patients > 70 years of age, respiratory rate, rale in lung auscultation, white blood cell counts, and ABG were extracted from patients’ records by a researchers at ICU for two consecutive days.

In case the patient attained at least one score from "signs and symptoms" and at least two scores from "other signs and symptoms" section of PNU1-CDC criteria (1, 7, 8), consultations with a pulmonologist subspecialist and radiologist (to interpret CXR) who were unaware of the study protocol and criteria assessment were requested. If interpretation of chest radiography was consistent with at least one score, diagnosis of pneumonia was confirmed according to the PNU1-CDC criteria. Accordingly, patients were divided in two groups: HAP positive (HAP^+^) and HAP negative (HAP^-^). Furthermore, an ICU-specialist physician who was not aware of the diagnosis based on CDC criteria was asked to evaluate the occurrence of pneumonia during daily visit for all patients independently. Physicians’ clinical judgment about presence of pneumonia was recorded based on pulmonologist subspecialist and/or ICU specialist who were unaware of the study protocol. Frequency of each CDC criterion was assessed. Frequency of the diagnosis of HAP was compared with physicians’ judgment. The data were transferred to the prepared checklists and subsequently to a SPSS v.19 (SPSS Inc., Chicago, IL, USA).

In order to analysis the data, mean of quantitative variables such as age, duration of hospitalization, and frequency of qualitative variables including sex were calculated. Kolmogorov-Smirnov test was used to assess the distribution of the variables. Mean of the quantitative variables between two groups were evaluated via independent-samples t test and categorical variables were assessed by Chi square and Fisher's exact tests. Correlations of variables were appraised by Pearson correlation coefficient. For each variable, 95% confidence interval (95% CI) was reported. Alpha level (P value) < 0.05 was considered as statistically significant.

All patients were informed that they were enrolled in a study and informed consent was signed by them. Patients’ information was kept confidential and was not provided to any organization for any reason. Neither extra charge nor invasive procedure was posed to patients.

## 4. Results

In this study, 300 patients who were admitted to Tehran heart center ICU after CABG were studied for a period of about six months from September 2011 to February 2012. All patients remained in the study for the whole study period and no patient was excluded from the study. Nine patients (3%) were diagnosed with HAP according to the CDC criteria; however, physicians who were unaware of the diagnosis according to the CDC criteria did not detect any case of pneumonia among the 300 studied patients. Frequencies of PNU1-CDC criteria for diagnosis of hospital-acquired pneumonia are summarized in four tables as follows: basic, laboratory, and clinical (Table 1), respiratory criteria (Table 2), ABG criteria (Table 3), and radiologic criteria (Table 4).

**Table 1.** Basic, Laboratory, and Clinical Criteria for Diagnosis of Hospital-Acquired Pneumonia ^a^

Variable	Overall Frequency (n = 300)	Hospital-Acquired Pneumonia		
		No	Yes	P value
**Sex**				
Male	215 (75)	219 (75)	6 (66.6)	0.44
Female	85 (25)	81 (25)	3 (33.3)	0.85
**Mean Age, y**	61.97 ± 9.62	61.91 ± 9.68	64.11 ± 7.59	0.49
**Intubation Time, min**	699.2 ± 326.6	691.5 ± 308.4	946.7 ± 685.4	0.02
**Duration of ICU Stay, d**	1.58 ± 0.84	1.58 ± 0.83	1.67 ± 1.00	0.75
**Age Range, y**	40-89	40-89	54-79	0.65
**Leukocytosis in 24 h**	159 (53)	155 (53.3)	4 (44.4)	0.42
**Leukocytosis in 48 h**	197 (65.7)	190 (65.3)	7 (77.8)	0.35
**Fever in 24 h**	1 (0.3)	1 (0.3)	0 (0)	0.97
**Leukopenia in 24 h**	5 (1.7)	5 (1.7)	0 (0)	0.85
**Leukopenia in 48 h**	3 (10)	2 (0.7)	1 (11.1)	0.08
**Loss of Consciousness in patients ≥ 70 Years Old in 24 h**	1 (0.3)	0 (0)	1 (11.1)	0.03
**Loss of Consciousness in Patients ≥ 70 Years Old in 48 h**	2 (0.7)	0 (0)	2 (22.2)	0.001

^a^ Data are presented as No. (%) or mean ± SD.

**Table 2.** Respiratory Criteria for Diagnosis of Hospital-Acquired Pneumonia ^a^

Variable	Total Frequency (n = 300)	Hospital Acquired Pneumonia Based on CDC Criteria		
		No	Yes	P value
**Respiratory Rate > 20/min in 24 h**	9 (3.0)	7 (2.4)	2 (22.2)	0.02
**Respiratory Rate > 20/min in 48 h**	4 (1.3)	1 (0.3)	3 (33.3)	< 0.001
**Dyspnea in 24 h**	10 (3.3)	7 (2.4)	3 (33.3)	0.002
**Dyspnea in 48 h**	7 (2.3)	4 (1.4)	3 (33.3)	< 0.001
**Worsening of Cough in 24 h**	1 (0.3)	1 (0.3)	0 (0)	0.97
**New-Onset Cough in 24 h**	6 (2.0)	5 (1.7)	1 (11.1)	0.97
**New-Onset Cough in 48 h**	5 (1.7)	4 (1.4)	1 (11.1)	0.142
**New-Onset Purulent Sputum in 24 h**	4 (1.3)	3 (1.0)	1 (11.1)	0.11
**New-Onset Purulent Sputum in 48 h**	8 (2.7)	7 (2.4)	1 (11.1)	0.21
**Increased Reparatory Secretions in 48 h**	3 (1.0)	3 (1.0)	0 (0)	0.91

^a^ Data are presented as No. (%).

**Table 3.** Blood Gas Analysis Criteria for Diagnosis of Hospital-Acquired Pneumonia ^a,b^

	Time of performing ABG	Frequency in total population (n = 300)	Hospital Acquired Pneumonia		
			No	Yes	P Value
**PaO**_**2**_ ** < 60 mmHg**	At ICU Admission,	1 (0.3)	1 (0.3)	0 (0)	0.97
	At 24 h After Admission	9 (3.0)	7 (2.4)	2 (22.2)	0.02
	At 48 h After Admission	49 (16.3)	43 (14.8)	6 (66.7)	< 0.001
**PaO**_**2**_ **/FiO**_**2**_ ** ≤ 240**	At ICU Admission,	247 (82.3)	238 (81.8)	9 (100)	0.16
	At 24 h After Admission	112 (37.3)	105 (36.1)	7 (77.8)	0.01
	At 48 h After Admission	178 (59.3)	170 (58.4)	8 (88.9)	0.06

^a^ Data are presented as No. (%).

^b^ Abbreviations: ABG, arterial blood gas; PaO_2_, partial pressure of oxygen in arterial blood; and FiO_2_, fractional concentration of inspired oxygen.

**Table 4.** Radiologic Criteria for Diagnosis of Hospital-Acquired Pneumonia ^a^

Variable	Frequency in total population (n = 300)	Hospital Acquired Pneumonia		
		No	Yes	P Value
**Diffused Infiltration in 24 h**	10 (3.3)	10 (3.4)	0 (0)	0.73
**Diffused Infiltration in 48 h**	6 (2.0)	6 (2.1)	0 (0)	0.33
**Localized Infiltration in 24 h**	43 (14.3)	42 (14.4)	1 (11.1)	0.62
**Localized infiltration in 48 h**	66 (22)	57 (19.6)	9 (100)	< 0.001
**Cavitation in 24 h**	1 (0.3)	1 (0.3)	0 (0)	0.97
**Pleural Effusion in 24 h**	133 (44.3)	127 (43.6)	6 (66.7)	0.15
**Pleural Effusion in 48 h**	152 (50.7)	146 (50.2)	6 (66.7)	0.26
**Consolidation in 24 h**	1 (0.3)	1 (0.3)	0 (0)	0. 97
**Pneumothorax in 24 h**	1 (0.3)	1 (0.3)	0 (0)	0.97
**Pneumothorax in 48h**	2 (0.6)	2 (0.7)	0 (0)	0.94
**Patchy Infiltration in 24 h**	2 (0.6)	2 (0.7)	0 (0)	0.94
**Patchy Infiltration in 48 h**	7 (2.3)	7 (2.4)	0 (0)	0.80

^a^ Data are presented as No. (%).

No age or sex differences were observed between patients with and without HAP. Mean hospitalization in ICU was 1.5 ± 0.8 days (range, 1-5). The mean of the intubation duration was 11.6 ± 5.4 hours (range, 3-43). The Age of participants ranged from 40 to 89 in HAP (+) group, from 54 to 79 in HAP (-) group (P = 0.65). Among laboratory and clinical criteria, the frequency of leukocytosis in 24 hours (P = 0.42), fever in 24 hours (P = 0.85), and leukopenia in 24 hours (P = 0.85) were higher in HAP (-) group in comparison with the HAP (+) group. Although the frequency of leukocytosis in 48 hours (P = 0.35) and leukopenia in 48 hours (P = 0.08) was higher in HAP (+) group, the difference was not significant.

Loss of consciousness in patients ≥ 70 years of age in 24 hours (P = 0.03) and 48 hours (P = 0.001) were significantly more frequent in HAP (+) group. Among respiratory criteria, worsening of cough in 24 hours (P = 0.97), and Increased respiratory secretions in 48 hours (P = 0.91) in HAP (-) group were more frequent than in HAP (+) group and new-onset cough in 24 hours (P = 0.97) and 48 hours (P = 0. 14) as well as new-onset purulent sputum in 24 hours (P = 0.11) and 48 hours (P = 0.21) were more frequent in HAP (+) group; however, the difference was not significant. Respiratory rate > 20/min in 24 hours (P = 0.02) and 48 hours (P ≤ 0.001) as well as dyspnea in 24 hours (P = 0.002) and 48 hours (P ≤ 0.001) were significantly more frequent in HAP (+) group. Among ABG criteria, PaO2/FiO2 ≤ 240 at ICU admission (P = 0.16) and in 48 hours (P = 0.06) were higher in HAP (+) group although the difference was insignificant. on the other hand, PaO2 < 60 mm Hg in 24 hours and 48 hours (P < 0.001) and PaO2/FiO2 ≤ 240 in 48 hours after surgery were significantly different between groups (P = 0.01). 

Among radiologic criteria, diffused infiltration in 24 hours (P = 0.73) and 48 hours (P = 0.33), localized infiltration in 24h (P = 0.62), consolidation in 24h (P = 0. 97), pneumothorax in 24 hours (P = 0.97) and 48 hours (P = 0.94), and patchy infiltration in 24 hours (P = 0.94) and 48 hours (P = 0.80) were more frequent in HAP (-) group. Although pleural effusion in 24 hours (P = 0.15) as well as 48 hours (P = 0.26) was more frequent in HAP (+) group, the difference was insignificant. In addition, localized infiltration in 48 hours (P ≤ 0.001) was significantly more frequent in HAP (+) group.

According to the PNU1-CDC criteria for the diagnosis of pneumonia, only two (0.7%) patients were identified with HAP in the first 24 hours of ICU admission; however; in the second 24 hours, nine patients (3%) fulfilled the criteria. All nine patients who were diagnosed with HAP according to the CDC criteria were discharged without any complication or need to antibiotic therapy in their hospitalization course.

## 5. Discussion

In this study, the presence of the CDC criteria (1, 3) was assessed in 300 patients undergoing CABG. As we know, this is the first study on evaluation of the frequency of clinical criteria of CDC in diagnosis of HAP and comparison of the detection of pneumonia according to CDC criteria and physicians’ judgment in patients undergoing CABG. This study showed that based on the PNU1-CDC criteria, 3% of patients were diagnosed with HAP while in none of the patients pneumonia was detected by physicians. This is the first study that provides quantitative and statistical evidence on the diagnostic criteria of HAP with regard to the clinical picture of the patient following CABG. We had a restricted inclusion criteria to avoid diagnostic problems in assessing the origin of symptoms, to distinguish new-onset from late-onset pneumonia-like symptoms and signs, and to avoid clinically doubtful cases. Considering the partially healthy group of patients in this study, it seems that real frequency of HAP as well as diagnosed HAP based on CDC criteria should be even higher in overall CABG or cardiac surgery patients.

Generally, clinical presentation of HAP is similar to the other types of pneumonia, which include fever, leukocytosis, increased respiratory secretions, and consolidation in physical examination, and changes in radiographic infiltration. Other clinical presentations may include tachypnea, tachycardia, decreased oxygenation, and increased minute ventilation (1, 7, 8). In intubated patients, due to high frequency of abnormal CXR findings prior to developing pneumonia and limitations of portable radiography technique, interpretation of radiography is troublesome (20).

Currently, no precise criteria exist for diagnosis of pneumonia in CABG patients, which highlights the findings of this study. Delayed or lack of detection of HAP seriously disturbs the efforts to prevent and treat HAP and can potentially lead to underestimation of the HAP importance in post-CABG mortalities. Routine application of PNU1-CDC criteria in such group of patients could lead to overdiagnosis of HAP.

It is reported that both quantitative culture and clinical approach are appropriate for evaluating the false-positive clinical diagnoses according to the recent guidelines of IDSA/ATS (2, 7). Our findings suggest that clinical approach detects more cases in comparison with the physicians’ judgment; therefore; to approve diagnosis, using PNU2 criteria is more justified than other group of patients, especially when there is some doubt about the diagnosis.Our findings suggest that clinical approach detects more cases in comparison with the physicians’ judgment. Simsek et al. studied prevalence of VAP in 1716 ICU-admitted patients who had undergone cardiac surgery. In their study, 36 patients (2.09%) were diagnosed with VAP according to CDC criteria with the mortality rate of 30% (23). These figures are lower than our observation, presumably due to the application of the quantitative CDC criteria in this study and/or restrictive inclusion criteria to rule out all complicated patients, which leads to a partially healthy study group. Furthermore, this study assessed prevalence of the pneumonia in patients undergoing major cardiac surgery but we focused on CABG.

Frequency of VAP was 7.87% in a study by Bouza et al. and central nerves system disturbances, mechanical ventilation > 96 hours, and re-intubation were associated with the development of respiratory infections (24). In this study, VAP mortality rate was 57.1%, tracheobronchitis was evident in 20.7%, colonization of bacteria was observed in 11.5%, and un-colonized patients constituted 1.6% of individuals, which were higher than the frequencies observed in our study. Previously mentioned suggestions could be applied here, too. Hortal et al. applied the revised CDC criteria for the diagnosis of VAP following cardiac surgery and found that VAT occurred in 3.7 cases per 1000 individuals undergoing mechanical ventilation (16). Therefore, frequency of HAP in our study was different from previous studies due to heterogeneity of the study protocols.

Our findings suggest that loss of consciousness in patients ≥ 70 years of age, tachypnea, dyspnea, PaO_2_ < 60 mm Hg, PaO_2_/FiO_2_ < 240, and localized infiltration in 48 hours were the criteria that contribute to different frequency of diagnosis between the PNU1-CDC criteria and physicians’ judgment; these could be misleading criteria and lead a probable overdiagnosis. It might be suggested that the value of these criteria for diagnosis of HAP in patients undergoing CABG and cardiac surgery patients should be revised, since presumably physicians do not solely judge based on CDC criteria and pay less attention to some of these criteria and consider the course of disease and interventions performed on patients.

On the other hand, leukocytosis in 24 hours and 48 hours, Fever in 24 hours, worsening of cough in 24 hours, new-onset purulent sputum in 24 hours, increased respiratory secretions in 48 hours, diffused infiltration in 24 hours and 48 hours, localized infiltration in 24 hours, pleural effusion in 24 hours and 48 hours, consolidation in 24 hours, pneumothorax in 24 hours and 48 hours, patchy infiltration in 24 hours and 48 hours were seen more frequently in HAP (-) group. Fever in 24 hours, leukocytosis and leucopenia in 48 hours, new-onset cough in 24 hours and 48 hours, new-onset purulent sputum in 24 hours and 48 hours, PaO_2_/FiO_2_ ≤ 240 at ICU admission and in 48 hours, and pleural effusion in 24 hours and 48 hours were more frequent in HAP (+) group; however, they were not significant might be more specific to HAP diagnosis in CABG. Notably, nine patients who were diagnosed with pneumonia according to the CDC criteria were discharged with no need of antibiotic therapy for pneumonia. This significant finding should be evaluated in details in further studies.

In conclusion, according the patients’ course of disease, expert physician may intentionally not take some of these criteria into account, consider some of them more important, or focus on those that are not included in criteria but give clues to diagnosis. Therefore, it is suggested that the value of these criteria should be re-evaluated in special group of patients such as CABG patients.

### 5.1. Limitations

Low sample size of this study, which was a consequence of the short period of the study and restricted inclusion criteria to avoid diagnostic conflicts, might lead to lower frequency of HAP detection. Further studies are needed to assess longer periods and more patients. Considering longer follow-ups of more than 48 hours might also be beneficial.

In order to limit the exposure of the patients to the X-ray, we only requested CXR for the patients who met the PNU1-CDC clinical criteria; radiologic evaluation of all patients seems necessary for more precise results. Considering the invasive nature of bronchoalveolar lavage (BAL), we assessed physicians’ judgment up to discharge of the patients. It seems rational to perform a larger multicenter study based on laboratory, clinical judgment, and prognosis of patients to assess the diagnostic value of those criteria, which had higher prevalence in HAP (-) group or were not significant in this study. Although there were limited HAP (+) patients, the results confirmed the idea of the study. More studies with larger sample sizes can confirm our conclusions. 
